# P185: The testing procedure of antimicrobial copper's Cu^+^ final product as a method of assurance and certification of its antimicrobial efficacy

**DOI:** 10.1186/2047-2994-2-S1-P185

**Published:** 2013-06-20

**Authors:** P Efstathiou, E Kouskouni, K Karageorgou, Z Manolidou, S Papanikolaou, M Tseroni, E Logothetis, C Petropoulou, V Karyoti

**Affiliations:** 1 National Health Operations Centre, Athens, Greece; 2 Medical School of the University of Athens, Microbiology Laboratory of Aretaieio Hospital, Athens, Greece; 3 “Agia Sofia” Childrens Hospital (NICU), Ministry of Health, Athens, Greece

## Objectives

The aim of this study is to record the testing procedure of antimicrobial copper's Cu ^+^ final products implemented in different facilities in order to reduce microbial flora.

## Methods

In areas where Cu^+^ [Intensive Care Unit (ICU) and schools] has already been implemented, random samples were collected for microbiological cultures using both wet and dry method (technique). The samples were collected in 3 different time - periods: during, 2 and 6 months after the implementation. All product manufacturing stages were recorded and taken into account by the construction company as well as maintenance and cleaning procedures of Cu^+^ surfaces and objects. Culture techniques in all samples collected were identical. 256 Cu^+^ surfaces and objects were tested and over 768 cultures for bacteria and viruses were taken, deriving all from 4 different facilities.

## Results

The antimicrobial effectiveness of the surveyed Cu^+^ objects and surfaces varied between 90-95%. Parameters such as multiple use, cleaning materials, conditions of humidity and dryness, appear not to affect the effectiveness of Cu^+^. The algorithm of the product testing procedure was recorded.

## Conclusion

The management of the facilities where Cu+ was implemented, demanded that the final Cu+ product was tested.

The testing procedure of the Cu^+^ final product was a requirement from the management whose facilities were implemented.

The above procedure is a method of implementations' assurance and certification, gives further value to the innovative implementation of antimicrobial alloys and ensures the possible side effects of distortion of the raw material and fake product manufacturing (FALSE).

## Disclosure of interest

None declared

